# Development of a zoomorphic test specimen for constancy testing on digital X-ray systems in veterinary radiology

**DOI:** 10.1186/s13028-019-0475-z

**Published:** 2019-08-20

**Authors:** Gerrit Pöhlmann, Matthias Lüpke, Christian Seiler, Patrick Wefstaedt, Jan-Peter Bach, Ingo Nolte, Hermann Seifert

**Affiliations:** 1 Institute for General Radiology and Medical Physics, University of Veterinary Medicine Hannover, Bischofsholer Damm 15, Haus 102, 30173 Hannover, Germany; 2 Small Animal Clinic, University of Veterinary Medicine Hannover, Bünteweg 9, 30559 Hannover, Germany

**Keywords:** 3D-print, Digital radiography, Image quality, Visual grading analysis

## Abstract

**Background:**

Technical failures and incorrect usage of digital X-ray systems may lead to a decreasing image quality, artefacts and a higher dose exposure of staff and patients. Although there are no regulations regarding constancy testing in veterinary radiology all operators are required to avoid unnecessary exposure. The aim of this study was to develop a reasonably inexpensive zoomorphic 3D-printed test specimen for constancy testing that allows the detection of changing image quality by visual analysis.

Primarily, a calibration curve of the attenuation factor of the 3D-printing material (ZP150) was determined. MATLAB converted every pixel value of a thorax X-ray image of a Beagle dog into an equivalent thickness of printing material. The thickness distribution was printed using a 3D-printer. This printed test specimen was additionally provided with five thin aluminium discs to simulate lung nodules.

To evaluate the usability for constancy testing 12 X-ray images of the test specimen were made. Two images (reference and control) were taken with the minimum dose in order to obtain images suitable for diagnosis purposes. Eight images were taken with a dose differing 30–140% from the reference dose by varying current–time product (mAs) or tube voltage (kVp). Two images were taken with the same parameters as the reference image but edited with different image processing. Six veterinarians (general practitioners) evaluated ten chosen structures in the X-ray images in a Visual Grading Analysis and scored the image quality of these structures for every image in comparison to the reference image. A Visual Grading Analysis Score was calculated and statistically analysed.

**Results:**

A higher current–time product led to a negligibly better evaluation of the X-ray image. The lower the current–time product the worse the X-ray images were scored. Likewise, both increasing and decreasing of the tube voltage led to lower scores.

**Conclusions:**

A zoomorphic test specimen can be used for constancy testing of digital X-ray systems in veterinary medicine. Especially a lower dose can be recognised due to deviation in the image quality when compared to the reference image. The 3D-printed test specimen is less expensive than test equipment used in human medicine.

## Background

X-ray examination is a common technique used in veterinary medicine, especially in small animal clinics. In the past years, more and more veterinary clinics have changed from using conventional radiography to digital radiography [1, 2]. Despite the fact that digital radiography offers numerous benefits, there are still some problems. Due to failure in X-ray equipment or human failure the image quality can decrease. Furthermore, artefacts can occur, so that additional pictures have to be taken and the radiation exposure of the patients and the staff can increase [1–5]. It is very difficult to recognise a slight decrease in image quality or a small increase in dose without the aid of a measuring instrument. Therefore, constancy testing is legally required in human radiology. These legal requirements should guarantee technically correctly adjusted X-ray equipment [6]. However, to date, there are no regulations concerning constancy testing in veterinary radiology in Germany and most other countries. Nevertheless, every operator of an X-ray device has to ensure that human radiation exposure and that of the environment are kept to a minimum [7].

The routine quality control testing of X-ray systems in Ireland during 2006 and 2007 revealed major or minor problems in 76% of the systems [3]. Most problems occurred with the automatic exposure control and the beam alignment, but also the dose output varied significantly [3]. Furthermore, a common problem with computed radiography is the use of higher exposure parameters than needed, which is known as “exposure creep” [8]. This leads to a higher radiation exposure of patients and staff [9]. All in all, these failures caused by human or machine error provide the risk of possible higher radiation exposure of staff and patients.

In Germany, the Guidelines on Radiation Protection in Veterinary Medicine (Strahlenschutz in der Tierheilkunde) is supposed to aid the user of an X-ray system to comply with the legal regulations of the Radiation Protection Law (Strahlenschutzgesetz) and the Radiation Protection Ordinance (Strahlenschutzverordnung (StrSchV)), respectively. However, there are no specific instructions for constancy testing [10]. Therefore, constancy testing of X-ray systems is more or less optional in veterinary radiology. Furthermore, the equipment for constancy testing used in human radiology according to DIN (Deutsches Institut für Normung—German institute for standardisation) 6868-13 [19] is relatively expensive (ca. € 3000—NORMI 13 Set PMMA X-ray test object and Conny II Dosimeter—Information given by PTW Freiburg GmbH on 01.06.2019) and the motivation for buying this equipment is low. The likelihood of veterinary surgeons performing a constancy testing on their X-ray systems without legal pressure is low.

The aim of this research study was to develop a reliable and reasonably inexpensive method for constancy testing on digital X-ray systems in veterinary radiology. Our hypothesis was that constancy testing in veterinary medicine should be feasible for laymen without complicated testing equipment. For this purpose, a zoomorphic phantom, which, if radiologically examined, almost looks like an ordinary X-ray image, was developed with a 3D-printer. After producing the phantom the usability of this phantom for constancy testing was investigated. Therefore, X-ray images either with different exposure parameters or different image processing were taken. The image quality of these X-ray images was compared with that of a reference image by means of a visual grading analysis (VGA). Using the results of the VGA, a method for constancy testing on digital X-ray systems using the phantom was to be developed. The presentation of this method shall provide a guideline for a voluntary constancy testing performed by veterinary surgeons to keep their X-ray systems running sufficiently and that should meet the principles of the StrSchV.

## Methods

Fiebich et al. [11] presented a method for producing an anthropomorphical phantom of the human breast with a 3D-printer. This method was used as a guideline for developing a zoomorphic phantom.

### Equipment

For all performed examinations two different pieces of X-ray equipment were used due to logistical reasons. For determining the attenuation characteristics of the 3D-print material and the correlation between tube voltage and dose the X-ray equipment the X-ray machine APR-Vet (Sedecal, Madrid, Spain) in the Institute for General Radiology and Medical Physics (University of Veterinary Medicine Hannover, Foundation) was used. The APR-Vet was used in combination with a Vita 25 computed radiography reader (Carestream Health GmbH, Stuttgart, Germany) and the software dicomPACS^®^ DX-R (Oehm and Rehbein GmbH, Rostock, Germany). The X-ray template of the test specimen and the X-ray images for the evaluation were taken during clinical routine in the Small Animal Clinic (University of Veterinary Medicine Hannover, Foundation) with an RO 1750 ROT 360 X-ray machine (Philips, Amsterdam, The Netherlands) on a CRMD 4.0 image plate (resolution 0.1 × 0.1 mm^2^; AGFA Healthcare GmbH, Bonn, Germany). The images were read out with the Digitizer CR-85 X (AGFA Healthcare) and processed by the MUSICA™ software of the NX-workstation (AGFA Healthcare).

All 3D-prints were performed by a ZPrinter450 (3DSystems, Rock Hill, South Carolina, USA). The print material used was the ZP150 (3DSystems), which mainly consists of plaster (Table 1).

**Table 1:** Chemical composition of the 3D-print material ZP150 (3DSystems GmbH, Rock Hill, South Carolina, USA) [12]

Component	Approximate amount in % of the weight
Plaster	< 90
Vinyl polymer	< 20
Carbohydrates	< 10

### X-ray attenuation of the print material

At first, the X-ray attenuation constancy over time of the ZP150 was determined. For this purpose, discs (diameter: 60 mm) of differing thicknesses (range 2.3–31.1 mm) were placed on a flat ionisation chamber (type 77335, PTW Freiburg, Freiburg) and the dose was measured for different tube voltages (range 50–80 kVp) and current–time products (20 or 40 mAs). These measurements were repeated three times with an interval of 3 months between each measurement. With the resulting data an attenuation curve of the material ZP150 was calculated and the constancy of the material tested.

### Conversion of the image information of a X-ray image into a material thickness distribution

The conversion of the image information of an X-ray image into a material thickness distribution has been described by Fiebich et al. [11]. In our study it was technically not possible to gain access to the raw data of the detector. Instead, it was necessary to use a calibration body made of the print material to convert the pixel values into a material thickness for each pixel. The stairs-shaped calibration body contained four levels with different heights (5.8; 15.8; 25.9 and 46 mm). The base area of the calibration body was 20 × 20 mm^2^.

**Fig. 1:**
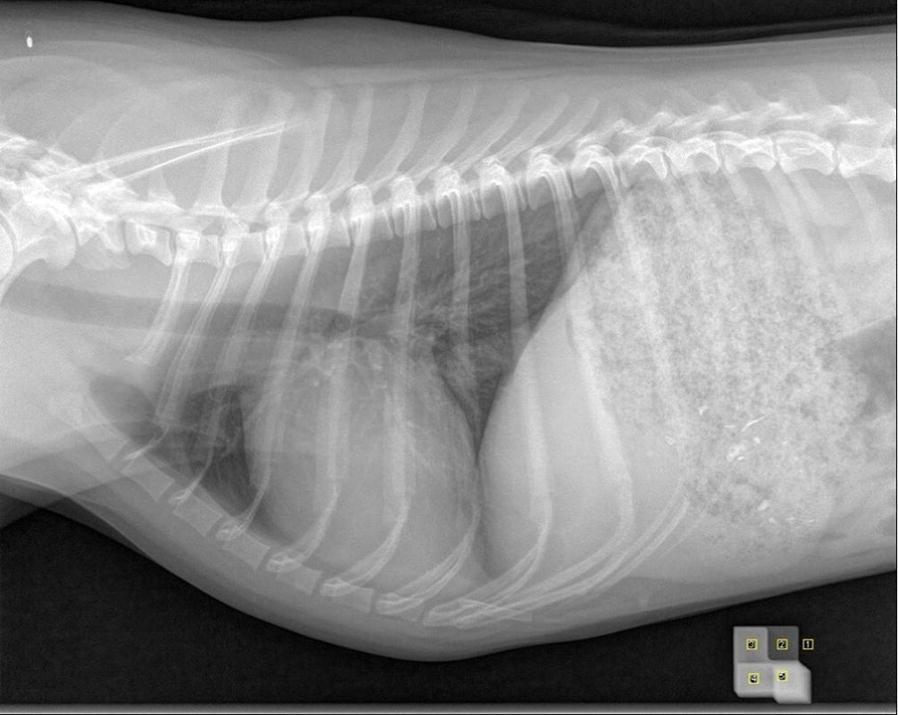
X-ray image of the thorax of the female beagle including the calibration body and the ROIs. The ROIs are the yellow squares placed on the calibration body and next to it in the bottom right corner of the figure

This calibration body was placed beside (ventral to) the abdomen of a female beagle during an X-ray examination of the thorax in a latero-lateral position. The X-ray examination was taken in line with a preventive medical examination for anesthesia. For the X-ray image a tube voltage of 60 kVp and a current–time product of 8 mAs (automatic exposure control) were chosen. The resulting X-ray image was loaded into the open source software ImageJ [13]. A region of interest (ROI) the size of 32 × 32 pixels was placed on every level of the calibration body and the average pixel value was measured for each level (Fig. 1). One ROI was moved towards the middle of the calibration body due to distortion effects caused by thickness of the calibration body and its location near the edge of the image. Additionally, a fifth ROI was placed next to the calibration for measuring a zero value. Using these five measured values a calibration function was computed describing the correlation between pixel value and material thickness.

**Fig. 2:**
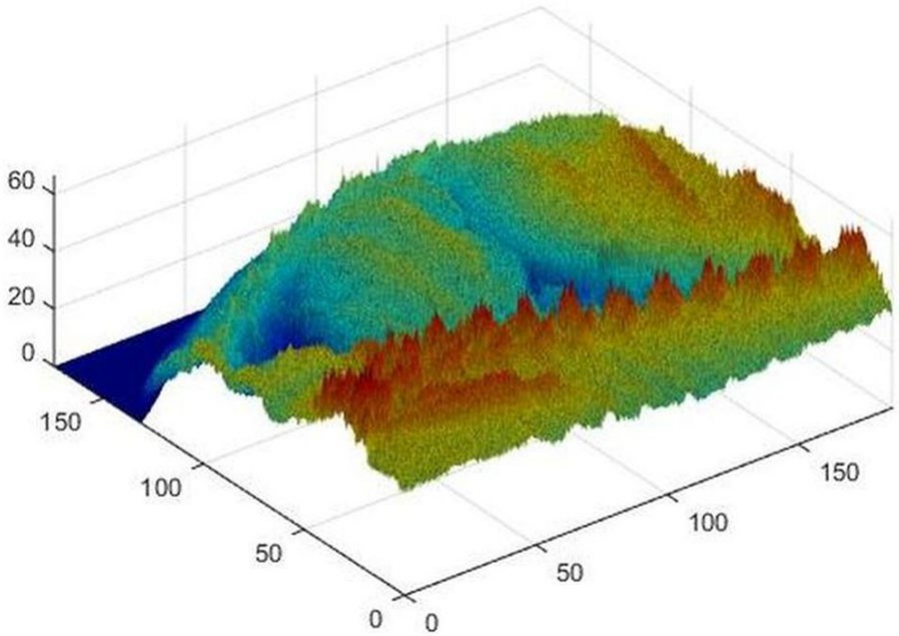
Thickness distribution of the material ZP150. Values of the axis labelling are written in mm. Control output from MATLAB

The image was loaded into MATLAB (MathWorks, Natick, Massachusetts, USA) and with a MATLAB script, which contains the calibration function, a material thickness was computed for each pixel of the X-ray image. The single values were combined to obtain a material thickness distribution in the stereolithography (STL) data format by MATLAB (Fig. 2). The surface of the material thickness distribution had been verified by the ZEditPro software (3DSystem) before being loaded into the printing software Zprint (3DSystems). The material thickness distribution was printed and afterwards the resulting three-dimensional test specimen was infiltrated with the glue Z-BondTM 90 (3DSystems), which makes the test specimen more resistant.

**Fig. 3:**
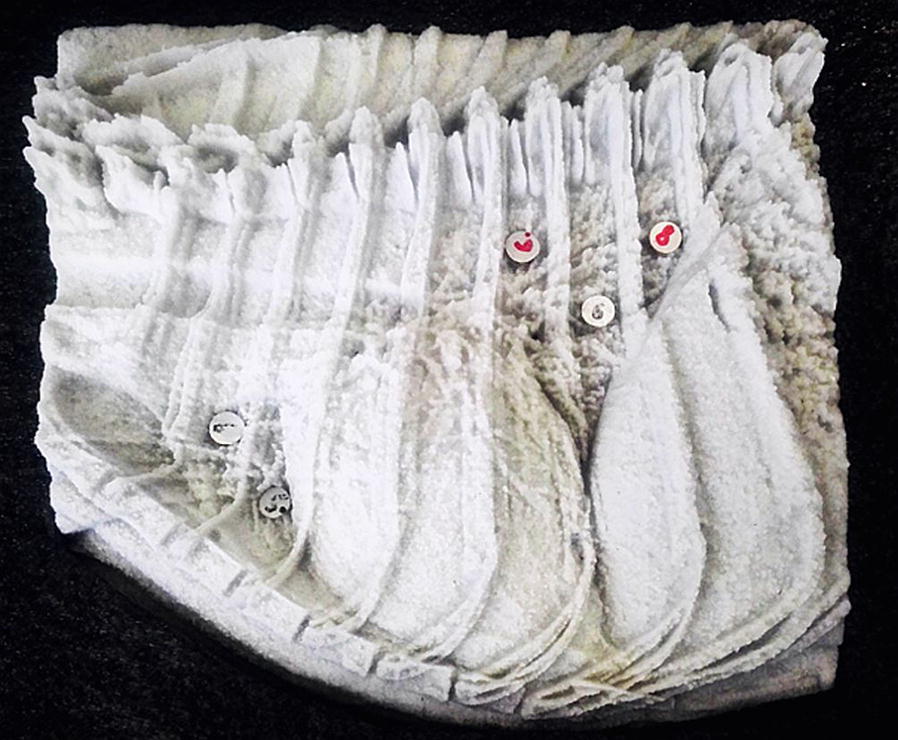
View on the printed test specimen (material ZP150) with the five added aluminium discs

Additionally, five aluminum discs (Diameter: 8.4 mm) of varying thickness (0.5–0.8 mm) were added as a modification to the test specimen (Fig. 3) to simulate lung nodules. The discs containing 99.5% aluminium were punched out of a solid aluminium plate (ALU-POINT GmbH & Co KG, Harsum, Germany). Thereafter, the discs were manually processed to a specified thickness in order to mimic the morphologic and hardly detectable appearance of lung nodules in X-ray images. According to Armbrust et al. [14], the appearance of lung nodules is fairly similar in all lungs. The five discs were spread among the cranial and caudal pulmonary lobes in the test specimen (Fig. 3).

### Evaluation of the usability of the test specimen for constancy testing

In order to evaluate the usability of the test specimen for the constancy test, 12 X-ray images of the test specimen were taken (Table 2). One image was taken with the standard dose in order to obtain an image suitable for diagnosis purposes and was used as a reference image. Another image was taken with the same exposure parameters as the control image. Eight images of the test specimen were taken, with the entrance dose differing from 30% to 140% from the reference dose. The dose was measured by placing the flat ionization chamber (Type 77335, PTW Freiburg, Freiburg) on the X-ray table. The relative dose changes induced by different current–time products (mAs) were calculated whereas the differences induced by different tube voltages (kVp) were measured with a flat ionisation chamber (Type 77335, PTW Freiburg, Freiburg) at the APR-vet X-ray machine. The intention of using alterations of the entrance dose and radiation quality was to simulate possible failures in the X-ray equipment. The alteration was either due to varying tube current–time product or tube voltage. The correlation between tube voltage and entrance dose had been determined previously experimentally. The two remaining X-ray images were taken with the same parameters as the reference image but edited with a different image processing. Instead of using the processing protocol for the thoracic soft tissue structures of small dogs (10 kg) in the lateral plane (protocol A), protocols for imaging bone structures of the head (protocol B) or abdominal soft tissue (protocol C) were used.

**Table 2:** Exposure parameters of the X-ray images

Image number	Tube current–time product [mAs]	Tube voltage [kV]	Entrance dose [%]	Protocol
1 (reference)	6.3	60	100	A
2	6.3	60	100	A
3	5	60	79	A
4	4	60	63	A
5	3.2	60	51	A
6	8	60	127	A
7	6.3	63	139	A
8	6.3	57	69	A
9	6.3	55	52	A
10	6.3	52	32	A
11	6.3	60	100	B
12	6.3	60	100	C

Images 11 and 12 were taken with a different image processing; protocol B (head) and protocol C (abdomen) instead of protocol A (thorax)

**Fig. 4:**
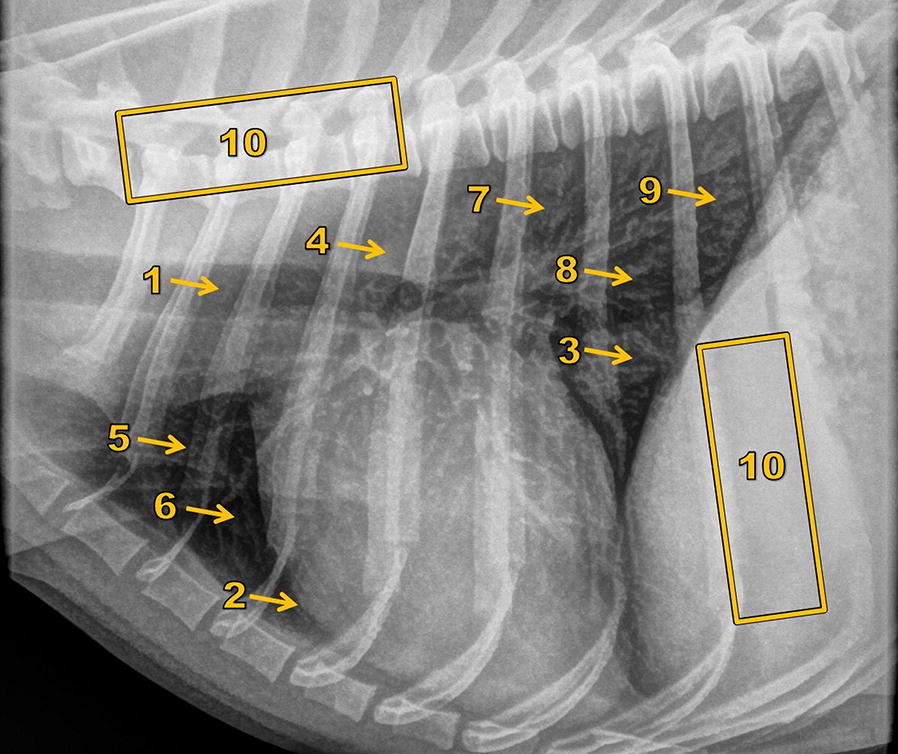
X-ray image of the test specimen showing the structures selected for the scoring. Structures selected for scoring: 1—trachea and proximal bronchia; 2—heart silhouette; 3—caudal vena cava; 4—thoracic aorta; 5—nodule 1; 6—nodule 2; 7—nodule 3; 8—nodule 4; 9—nodule 5; 10—regions for scoring the quantum noise impression

In the modified test specimen, four anatomical and five pathological structures (artificial nodules) were chosen (Fig. 4), which were to be scored by general veterinary surgeons who frequently assess thoracic X-ray images with regard to contrast, sharp contour and quantum noise impression throughout a visual grading analysis (VGA). Furthermore, the quantum noise impression of the image itself was to be scored in two different regions.

VGA is a method used to evaluate the image quality [15]. It has been shown before that the results of a VGA correlate with physical measurement for image quality [16, 17]. By performing a relative VGA the X-ray images 2–12 (Table 2) were compared with the reference image which was always visible for a side-by-side comparison. The order of the images was randomised. The proper function of the monitor was checked with a homogeneity check. The ambient light of the room was set to 25 lx. Before the real scoring was performed the veterinary surgeons had complete a training round with three slightly different X-ray images of the test specimen. For the VGA, the six veterinary surgeons scored the 11 X-ray images of the test specimen against the reference image. They compared all nine structures and the quantum noise impression alone on a 7-step scale (− 3, − 2,− 1, 0, 1, 2, 3). A score of − 3 means a far worse presentation of the structure, a score of 0 a pretty equal presentation and a score of 3 a much better presentation (Table 3). A visual grading analysis score (VGAS) was calculated from the scores of the six veterinary surgeons for each X-ray image using the following formula, which was described by Tingberg and Sjöström [18] and modified for this study accordingly:

$$ VGAS = \frac{{\mathop \sum \nolimits_{o = 1}^{{N_{O} }} \mathop \sum \nolimits_{s = 1}^{{N_{S} }} G_{s,o} }}{{N_{O} \times N_{S} }} $$

G_s,o_ is the individual score of an observer (O) for the structure (S) in a specific X-ray image. N_S_ is the total number of structures (N_S_ = 10), which are scored in an X-ray image and N_O_ is the total number of observers (N_O_ = 6).

**Table 3:** Verbalised scores of the visual grading analysis (VGA)

Score	Impression of the structure
+3	Much better presentation
+2	Better presentation
+1	Slightly better presentation
0	Equal presentation
− 1	Slightly worse presentation
− 2	Worse presentation
− 3	Far worse presentation

### Statistical methods

The resulting data were analysed with descriptive methods as well as with significance tests. A paired t-test was performed to investigate whether the scores (VGAS) of an X-ray image differed from the reference image. Furthermore, the same test was used to assess if some structures had a greater impact on the overall score (VGAS) than other structures. A result was considered significant when P < 0.05.

## Results

### X-ray attenuation of the print material

**Fig. 5:**
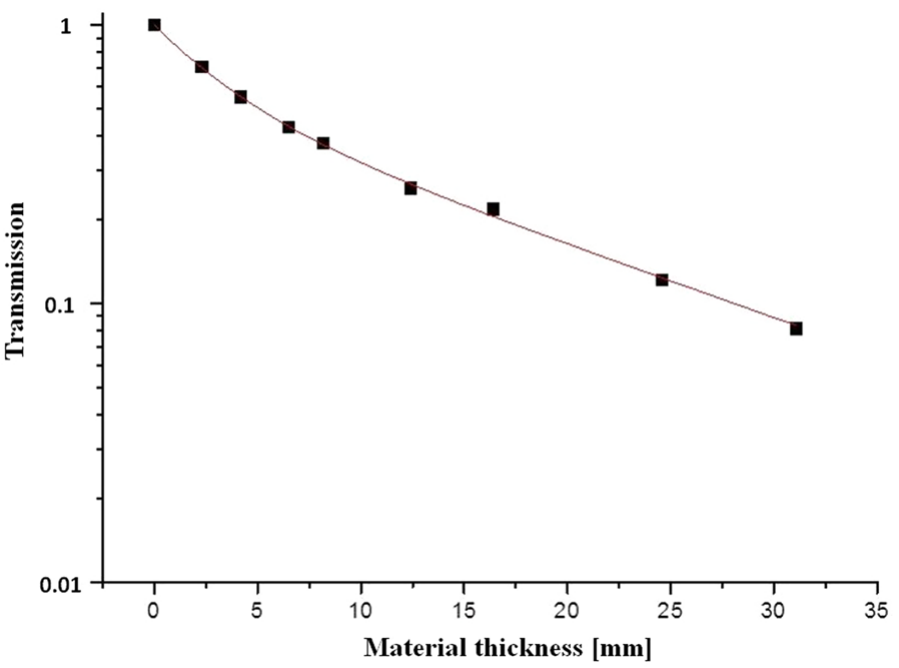
Attenuation curve of the print material ZP150 (3DSystems GmbH). The transmission is defined as follows: ${\text{T}} = \frac{{{\text{KERMA}}_{\text{x}} }}{{{\text{KERMA}}_{0} }}$ with ${\text{KERMA}}_{0}$: no material between X-ray tube and ionisation chamber and ${\text{KERMA}}_{\text{x}}$: x mm material between X-ray tube and ionisation chamber

The attenuation curve of the print material ZP150 approximately fitted an exponential function (Fig. 5). The differences of the measured values of all four temporally following measurements were minimal and there was no trend indicating a change in the attenuation properties of the material.

### Result of the 3D-print

**Fig. 6:**
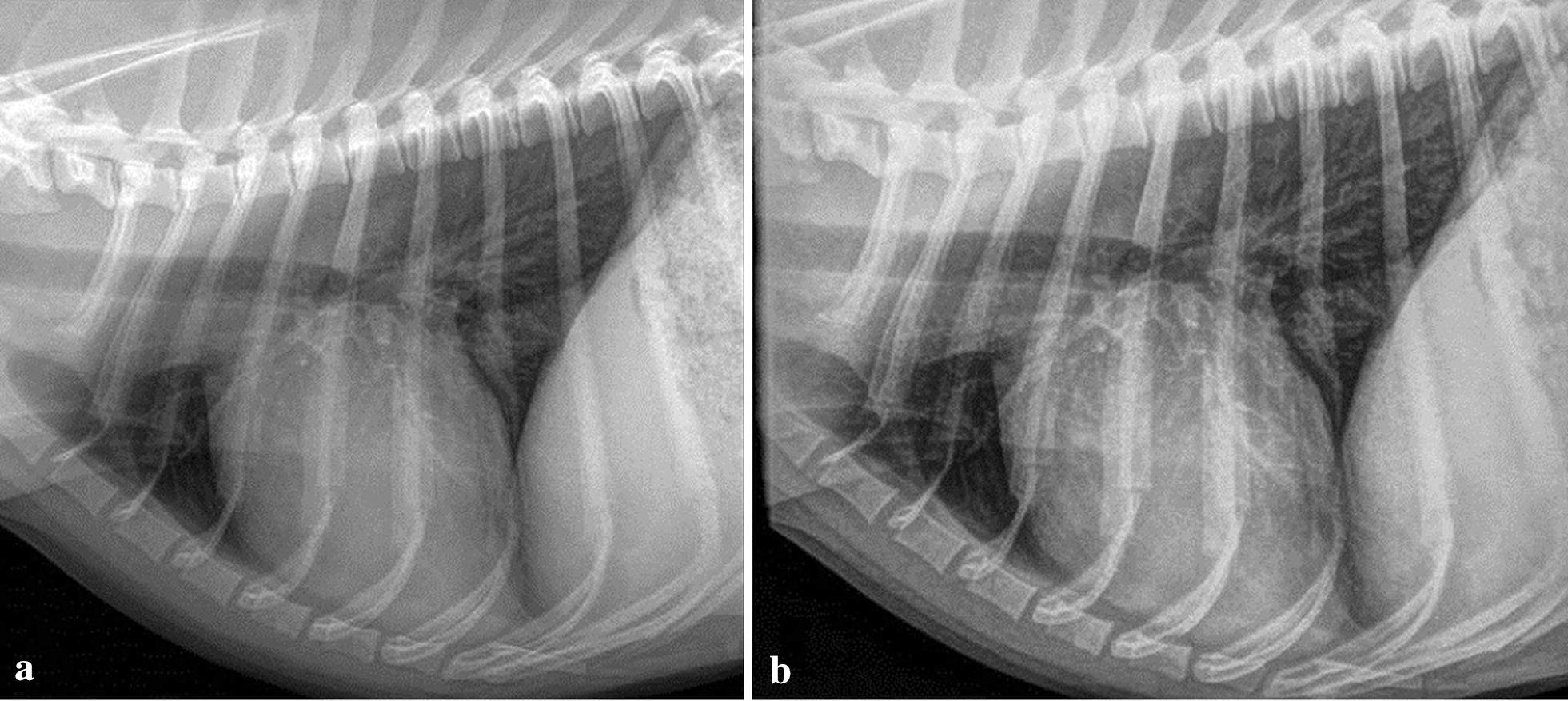
Comparison of the X-ray template (**a**) and an X-ray image of the test specimen (**b**)

The printed test specimen is shown in Fig. 3. It weighs 1586 g and is 18.6 × 17.4 × 6.6 cm^3^ in size. A comparison between the X-ray image of the female beagle, which is the template of the test specimen, and an X-ray image (60 kVp; 6.3 mAs automatic exposure control) of the test specimen is shown in Fig. 6. There are small differences in contrast, brightness and detail detectability. Especially the bronchial tree is more detailed in the original X-ray image of the female beagle. There is however, a strong compliance between the X-ray image of the female beagle and the X-ray image of the test specimen.

### Evaluation of the X-ray images of the test specimen

The analysis of the evaluation included 660 scores by the six veterinary surgeons. Table 4 shows a cross table of the mean values of the scores for every X-ray image and every structure. The mean values of the caudal vena cava varied slightly (± 0.5) around the zero value of the reference image. The mean values of the aorta, the heart silhouette and the trachea, together with the proximal bronchia differed a little more from the zero value of the reference image. The greatest deviations in the mean values in comparison with the reference image were found in the scores of the nodules 3 to 5 and the quantum noise impression. Additionally, in this group the scores of the nodules 3 and 4 showed the maximum deviation in the reference image. The mean values of the scores of the nodules 1 and 2 like the score of the caudal vena cava showed only small variations. Looking at the mean values (VGAS) of the complete X-ray images, images 4 and 5 showed the greatest deviation in comparison to the reference image. Only small deviations could be found in the VGAS of images 2 and 6.

**Table 4:** Mean values of the scores of the structures in all images (VGAS)

Image	1	2	3	4	5	6	7	8	9	10	11	12	Mean
Trachea and prox. bronchia	/	0.17	− 0.17	− 0.33	*− 1.50*	0.33	− 0.83	− 0.83	− 0.67	0.00	0.17	0.83	− 0.26
Heart silhouette	/	0.17	− 0.67	− 0.33	− *0.83*	0.17	0.33	− 0.17	*− 0.67*	0.50	0.33	0.00	− 0.11
Caud. V. cava	/	0.33	− 0.50	0.00	− 0.50	0.17	0.17	− 0.33	0.50	0.33	0.00	0.00	0.02
Thoracic aorta	/	− *0.83*	− 0.33	− 0.50	− 0.50	− 0.50	*− 0.67*	0.00	0.00	− 0.17	− 0.17	− 0.67	− 0.39
Nodule 1	/	− 0.17	0.17	− 0.67	− 0.50	0.17	0.17	0.33	− 0.33	*0.67*	− 0.33	0.00	− 0.05
Nodule 2	/	− 0.17	− 0.33	− 0.50	− 0.33	*0.83*	− 0.50	0.17	*0.67*	0.00	0.00	− 0.33	− 0.05
Nodule 3	/	− 0.67	− 0.33	*− 1.50*	*− 2.00*	0.50	− *1.00*	− *0.83*	*− 1.67*	*− 1.33*	*− 1.50*	− 1.00	− 1.03
Nodule 4	/	− 0.17	*− 1.50*	*− 1.50*	*− 2.17*	*− 1.17*	*− 1.67*	*− 1.17*	*− 1.50*	*− 0.83*	*− 1.33*	*− 1.33*	− 1.30
Nodule 5	/	0.17	*− 0.83*	− 0.83	− 0.50	0.17	*− 0.83*	0.00	0.00	− 0.50	0.00	− 0.67	− 0.35
Noise impression	/	− 0.17	− 0.50	− 0.67	*− 1.33*	− 0.17	− 0.17	0.00	− 0.67	*− 1.17*	− 0.17	− 0.17	− 0.47
Mean (VGAS)	/	− 0.13	− 0.50	− 0.68	− 1.02	0.05	− 0.50	− 0.28	− 0.43	− 0.25	− 0.30	− 0.33	

Italics values showed a significant different VGAS compared to the reference image due to the 5% significance level

### Visual grading analysis scores differentiated according to the dose and the image processing

**Fig. 7:**
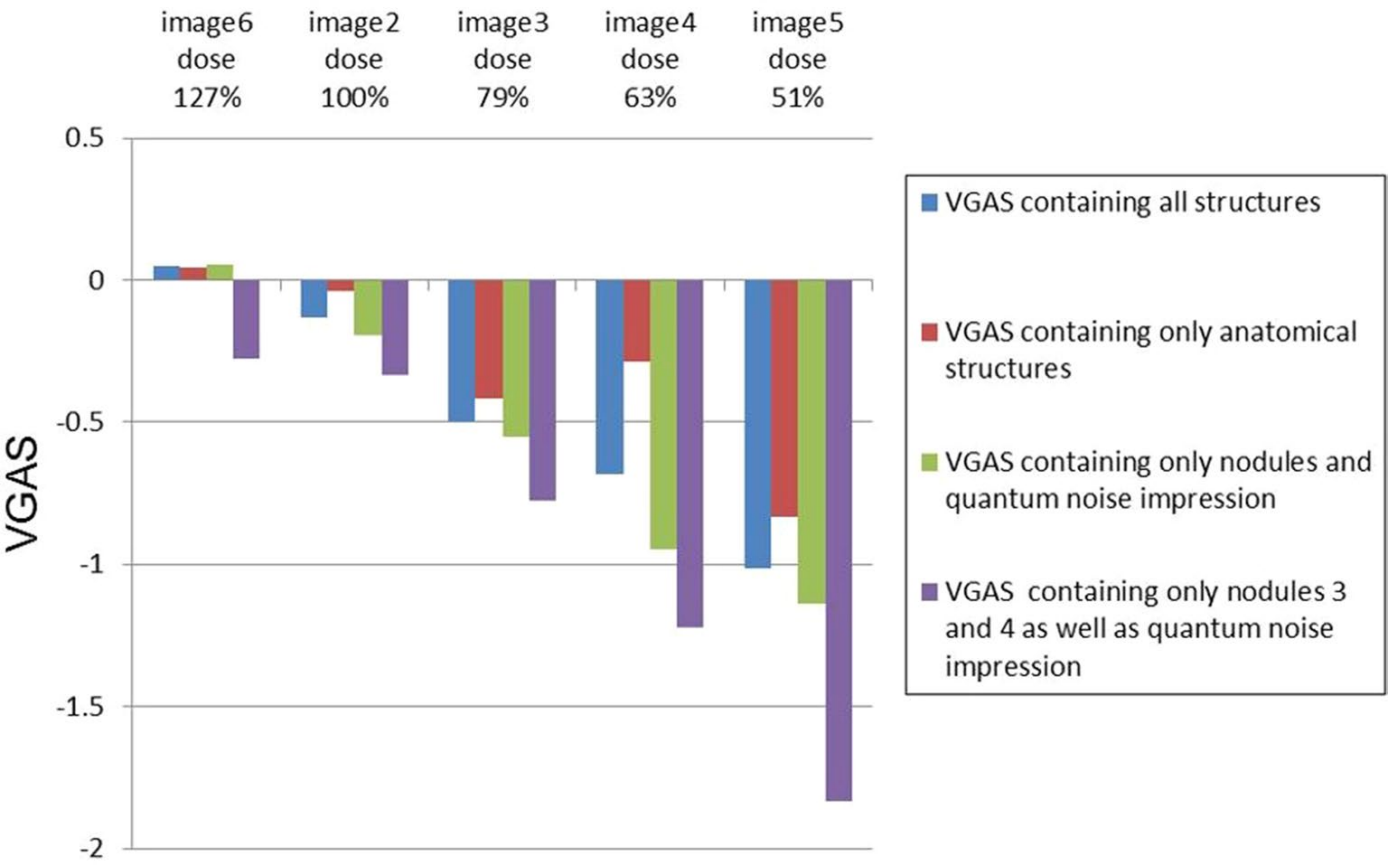
Bar graph of the VGAS of the X-ray images, which were taken with varying current–time products (mAs). The images are categorised by dose

The results of the scoring of the X-ray images with dropping dose due to varying current–time product (mAs) are shown in Fig. 7. The VGAS of the X-ray images containing all structures decreased with the falling current–time product (mAs). When the VGAS only contained the anatomical structures, the VGAS still decreased but less strongly. When only taking the nodules and the quantum noise impression into account the VGAS became more negative. When only looking at nodules 3 and 4 as well as the quantum noise impression the VGAS became even more negative. The X-ray image 6, which was taken with a higher dose (127% of the reference dose), got a slightly but not significantly higher VGAS than the control image, which was taken with the same X-ray parameters as the reference image. However, the VGAS containing the noise impression and nodule 3 and 4 were scored worse that the reference image which mainly results from the negative score of nodule 4. A possible explanation for that could be psychological effects as the observers mainly dealt with images of worse quality than the reference image and nodule 4 seems to be the structure that was the hardest to detect. So, maybe, the observers projected this hard detectability into a bad image quality.

**Fig. 8:**
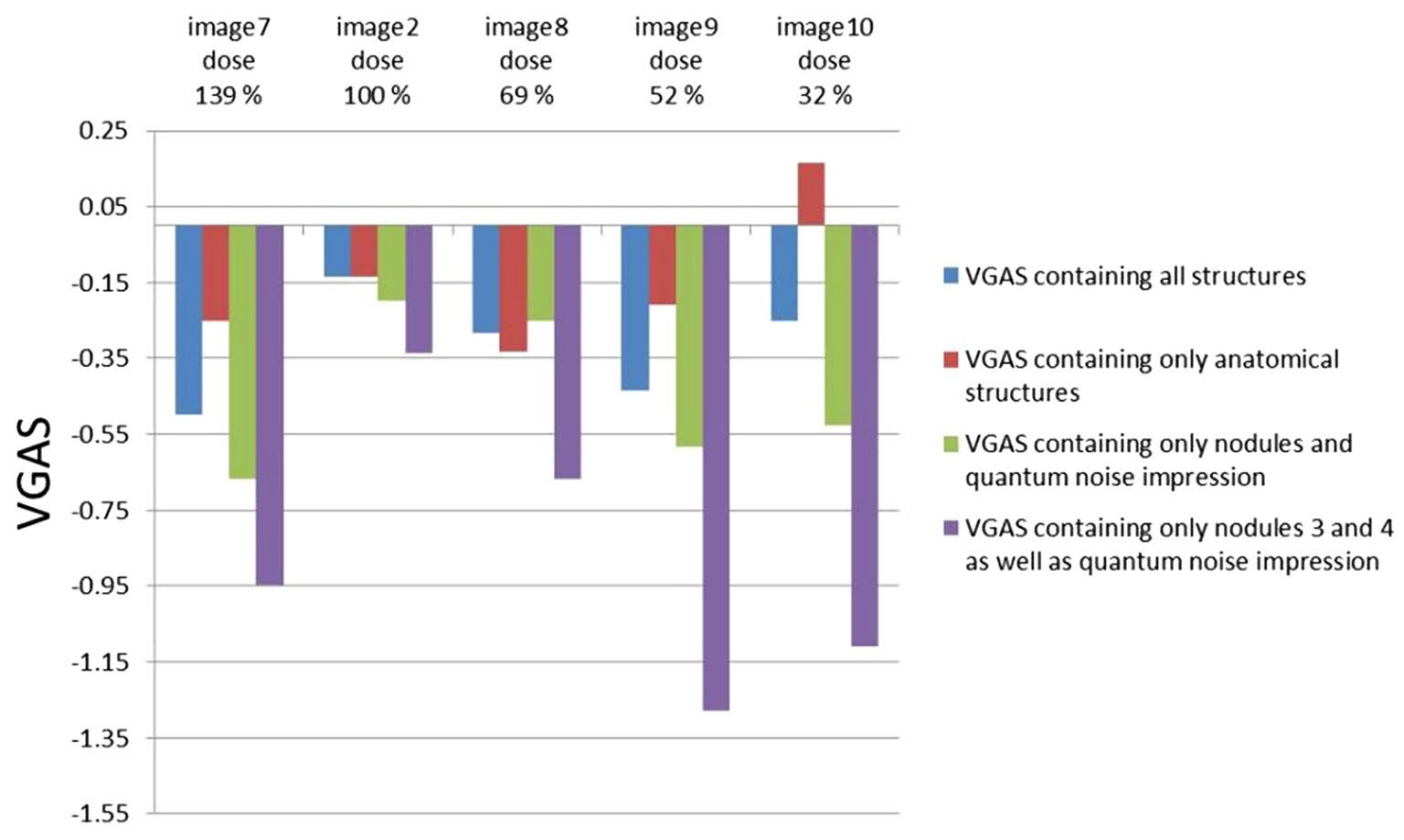
Bar graph of the VGAS of the X-ray images, which were taken with varying tube voltage. The images are categorised by dose

The results of the scoring of the X-ray images, which were taken with varying tube voltage and therefore varying dose, are shown in Fig. 8. Image 9 (relative dose 52%) had the lowest VGAS of all images taken with varying tube voltage. The VGAS increased slightly from image 9 to image 10 (relative dose 32%) although the dose decreased. When the VGAS only contained the anatomical structures, no trend could be determined. However, image 10 showed a slightly better VGAS than the reference image 2. This is surprising as the dose was reduced for image 10. In some cases a reduction of the tube voltage can enhance the contrast for some structures due to the lower energy of the radiation used. When the VGAS only contained the nodules and the quantum noise impression the scores for all images were worse in comparison with the VGAS containing all structures. The images had the worst VGAS when only the nodules 3 and 4 as well as the quantum noise impression were taken into account. Image 7 was taken with higher tube voltage (relative dose 139%) and had a worse VGAS compared with image 2 (control image) regardless of which structures were considered.

**Fig. 9:**
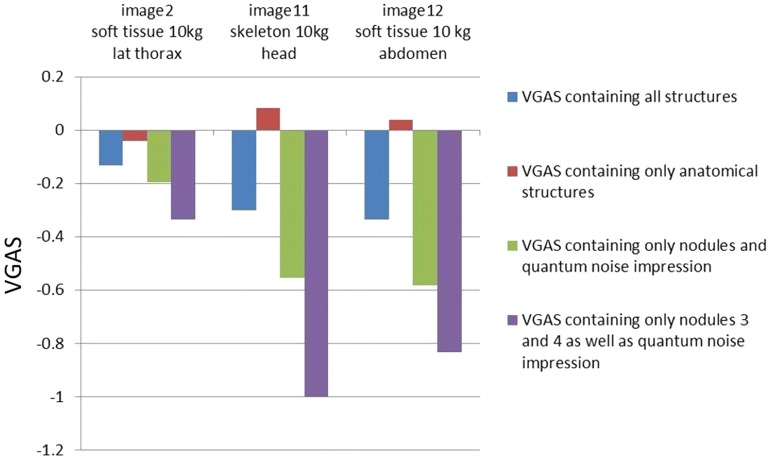
Bar graph of the VGAS of the X-ray images categorised by image processing

The results of the scoring of the X-ray images, which were taken with different image processing, are shown in Fig. 9. The VGAS containing all structures of the X-ray images, which differed in terms of image processing from the control image (protocol A), became worse. When the VGAS only contained the anatomical structures the images with the processing protocol B (image 11) and protocol C (image 12) scored slightly better. When the VGAS only contained the nodules and the quantum noise impression, or rather only nodules 3 and 4 as well as the quantum noise impression images 11 and 12 scored considerably worse.

### Results of the statistical analyses

The paired comparison of the scores of the structures in an image compared with the same structure in the reference image resulted in few differences at the 5% significance level. The most significant differences were computed for nodule 3 (seven differences) and nodule 4 (ten differences). The other structures differed only one or two times significantly from the same structure in the reference image. The caudal vena cava did not differ significantly at all and the quantum noise impression only twice.

The results of the paired comparison of the VGAS of the X-ray images using the paired t-test are shown in Table 5. For all X-ray images, except for images 2 and 6, statistical significant differences in comparison to the reference image were found.

**Table 5:** Results of the paired t-test

No. of X-ray image	Entrance dose change [%]	Tube current–time product [mAs]	Tube voltage change [kVp]	P-value from paired t-test	Protocol
2	0	6.3	0	0.185	A
3	− 21	5	0	< 0.001	A
4	− 37	4	0	< 0.001	A
5	− 49	3.2	0	< 0.001	A
6	+ 27	8	0	0.678	A
7	+ 39	6.3	+ 3	< 0.001	A
8	− 31	6.3	− 3	0.012	A
9	− 48	6.3	− 5	0.002	A
10	− 68	6.3	− 8	0.038	A
11	0	6.3	0	0.013	B
12	0	6.3	0	0.024	C

P-values of the paired t-test for the paired comparison between the VGAS of the reference image (1) and the images 2–12. The null hypothesis is that there is no difference between the VGAS. Image 11 and 12 were taken with a different image processing; protocol B (head) and protocol C (abdomen) instead of protocol A (thorax)

## Discussion

According to the results of the present study, the material ZP150 is well suited to be used for the development of a radiologic test specimen. The attenuation of the material is neither too high nor too low, so that the test specimen could be developed with a suitable thickness. Within the study period, no differences could be measured between the single measurements of attenuation characteristics of the ZP150. Therefore, a decrease in the image quality during constancy testing is not caused by a change in the attenuation characteristics of the print material.

The comparison between an X-ray image of the test specimen and the X-ray image of the thorax of the female beagle shows that the manufacturing method worked properly. The small differences are most likely caused by four factors: The first factor refers to the use of pixel values of the image of a calibration body instead of using the detector dose values like in the study of Fiebich et al. [11], which leads to an ambiguity between pixel value and dose. The second factor refers to the differences in pixel size (0.1 × 0.1 mm^2^) of the used image plate and the dot size (0.08 × 0.06 mm^2^) of the 3D-printer. As a result, the pixel and the print dot are slightly shifted, which may lead to a different presentation of small details in the images. The third factor refers to the image processing. After exposure of the thorax of the female beagle and the test specimen the raw data are transmitted to the MUSICA™ software and processed. Although the raw data are quite similar, small differences can lead to different presentation in terms of contrast and brightness. The fourth factor refers to the quantum noise, which can differ from picture to picture.

The simulation of lung nodules using aluminium discs worked properly. In the test specimen for constancy testing aluminium discs are used for determining the contrast resolution [19]. The discs used for the phantom in the present study were manually processed and therefore they had some little thickness variations. The aim of the aluminum discs was to simulate nodules which are difficult to detect and with which a deterioration of the image quality is noticeable. For this purpose, nodules 3 and 4 proved to be ideal, while the other nodules seemed to be easy to detect even with poorer image quality and thus did not generate significantly worse evaluations (Table 4).

At the visual rating of the X-ray images by means of a VGA the six veterinary surgeons usually scored the image quality of the images on the basis of the exposure parameters as would have been expected. The trend concerning whether a structure or an image was considered good or bad was alike but differences appeared in the scoring of the structures. A simultaneous training with all veterinary surgeons before the VGA might have resulted in an even better agreement.

The VGAS for the X-ray images which were made with a lower current–time product (mAs) (images 3–5) had the lowest values. Especially the VGAS of lung nodules 3 and 4 were scored the worst (Table 4). The DIN for constancy testing allows deviations in the dose in a range of ± 30% [19]. In our study due to limited adjustment options it was not possible to create an image with the exact dose of 70% but with 63% of the reference image (image 4). The anatomical structures were also graded worse with a lower dose, but not in the same manner as the nodules (Table 4). The nodules simulate low contrast structures. With these structures quantum and anatomical noise are much more prominent and noticeable than with larger structures which naturally have better contrast [20–22]. These results show that it is possible to recognise a dose drop to 70% of the reference dose through visual grading of structures (nodules) of the test specimen. The fact that a decrease in dose to 79% of the reference dose due to a lower current–time product (mAs) led to a bad presentation of the nodules 4 and 5 has a direct impact on the clinical practice. This is due to the fact that already a mAs-induced dose change of 20% can reduce the diagnostic sensitivity for the detection of nodules in the lung during X-ray examinations. This can have considerable consequences for the patient. For example, metastases in the thorax could be overlooked by the veterinary surgeon. Interestingly, concerning the noise impression the two images taken with a higher dose (images 6 and 7) were rated slightly worse than the reference image (image 1). However, the difference is by far not significant (P = 0.70 and P = 0.61) and is probably due to psychological effects, as the reviewers mainly had to evaluate images of poorer quality and subconsciously regarded the reference image as the optimum.

The results for the tube voltage-related dose changes reveal that a variation of the tube voltage (kVp), no matter if increasing or decreasing, leads to lower VGAS scores and a deterioration of the image quality. However, the differences are more moderate than those for mAs product-related dose changes. At X-ray image 10 (relative dose = 32%), the VGAS and therefore the presentation of the anatomical structures in image 10 even increases slightly compared to the reference image (Fig. 8). These, at first glance, somewhat contradictory results are due to an improvement in contrast due to the increasing photoelectric effect at a lower tube voltage. Although quantum noise increases at a lower dose, the improvement in contrast compensates for or overcompensates for degradation of image quality by increasing quantum noise. This hypothesis is supported by the fact that image 7 (relative dose = 139%), where mainly nodules 3-5 were rated very badly, was given a relatively bad VGAS (− 0.50). By increasing the tube voltage, the already low contrast between the nodules and the anatomical surroundings became even lower, resulting in a poorer presentation of the structures. As the entrance dose measurements were carried out at the APR-vet X-ray system and the images of the test specimen at the ROT 360 X-ray system the measured relative doses changes for different tube voltages will not be completely transferrable but in the authors opinion accurate enough to draw this comparison.

The results of the VGAS for the X-ray images with changed lookup table settings are not conclusive. On the one hand, the VGAS for the nodules and the quantum noise impression became worse (Fig. 9), On the other hand, the anatomical structures were rated relatively similarly in comparison to the reference image by the six observers. Specific look-up tables for different body regions should ensure that X-ray images are displayed optimally for these different indications [23]. Assumptions concerning the cause of the small differences in the presentation of the anatomical structures are difficult because the mathematical algorithms of the MUSICA™ software are not known in detail because of the trade secret.

In the hypothesis tests some restrictions had to be accepted due to the ordinal data structure of the visual grading analysis. When dealing with ordinal data, only non-parametric tests may be used. Norman [24], however, showed that it is possible to use parametric test methods for ordinary data without getting erroneous results. Similar studies [16, 18, 25] also work with parametric test procedures. Therefore the parametric paired t-test was chosen for analysing the ordinal data.

When the VGAS of the complete X-ray images is statistically analysed by paired t-test, the altered image quality is also visually well recognised. All X-ray images, except for images 2 (same parameters) and 6 [27% higher current–time product (mAs)], showed statistically significant differences in VGAS in comparison to the reference image. The X-ray images created with a lower current–time product (mAs) or an altered tube voltage showed statistically significant differences in the hypothesis tests. Accordingly, by evaluating visual images of the test specimen it is possible to detect changes in image quality caused by alterations in current–time product (mAs) and or tube voltage.

Unnoticed manual dose changes in X-rays do also occur with digital radiography. Increasing the dose in X-rays, the so-called “exposure creep”, can lead to increased radiation exposure for the medical personnel and the patient [9]. In addition, failures in the X-ray system can lead to deviations between the settings of the exposure parameters and the actual exposure parameters used [3]. For radiation protection reasons, X-rays should be taken in compliance with the ALARA (as low as reasonably achievable) principle [20]. In veterinary medicine, in contrast to human medicine, in addition to the patient, usually two staff members, who restrain the animal, are exposed during an X-ray examination. Therefore, especially in veterinary medicine it is important to perform constancy testing and quality assurance in digital radiography for reasons of radiation protection. No special legal regulations referring to constancy testing exist for veterinary medicine, but the German Guidelines on Radiation Protection in Veterinary Medicine (Strahlenschutz in der Tierheilkunde) [10] demands records concerning periodical function testing and service of X-ray systems. If a veterinary practice wants to apply for a GVP-certification (Good Veterinary Practice—Gute Veterinärmedizinische Praxis) the compliance with the existing regulations is checked. With regard to quality assurance in X-ray diagnostics, however, no further actions are required.

The results of the present study allow two statements. On the one hand, an incorrect exposure in digital X-ray examination was noticeable in the image quality in this research study. This could be seen in both mAs-induced underexposure and tube-voltage-related dose and contrast changes of the X-ray images. On the other hand, an overexposure did not necessarily lead to a better image quality. With a higher current–time product (mAs) or tube voltage (kVp) the noise impression in particular was scored slightly worse than in the reference image. Therefore an “exposure creep” cannot be identified specifically with this method. It should be borne in mind that any image quality degradation in chest X-ray images results in lower diagnostic sensitivity due to the complexity of the thorax and the variety of structures [9]. Also, changed lookup table settings of the image processing software can lead to image quality degradation. This can occur in particular if users of the X-ray device change the settings of the lookup table without consulting the manufacturer. On the other hand, the method developed for constancy testing in this study can in part detect the dose changes as required in the corresponding DIN [19].

A regular constancy testing of the X-ray system is especially important for larger clinics as there are many X-rays performed and the system is more stressed. Such a quality assurance could be performed at regular intervals (e.g., monthly) similar to constancy testing in human medicine. During commissioning of the X-ray system a reference image of the test specimen should be made with defined exposure parameters. Once a month, an X-ray of the test specimen should be made with the same exposure parameters, the same image plate and the same image processing and compared with the reference image. On the X-ray of the test specimen specific structures (e.g., nodules 3 and 4 of the test specimen) should be compared with those on the reference image and the results of these tests should be recorded. If there are severe deviations in the image quality, another X-ray of the test specimen should be made. If the second X-ray also shows a severe deviation in the image quality a systemic troubleshooting should be started. In case of unsuccessful troubleshooting, the operator of the X-ray system should contact the manufacturer in order to determine the exact cause of the problem and have the issue solved.

Jimenez et al. [1] list in their work a large number of different artefacts that can occur during digital radiography. Although this study does not specifically deal with the detection of artefacts, the VGAS method offers basically the possibility to detect a deterioration of the image quality due to artefacts as well. A complete quality assurance also includes the inspection of the correct function of the collimation. This was not investigated in this study with the test specimen. For this purpose, the test specimen would have to be further developed and mounted on a plexiglass plate which is slightly larger than the specimen and has markings at the corners made of wire, which can be seen in the X-ray image.

A test specimen, as it was developed in this study, would have costs of manufacture of about 400 € and would thus be well below the price of the corresponding test equipment used in human medicine, costing about 3000 €.

The results of this study show the suitability of the developed test specimen for constancy testing in veterinary digital radiography. However, a further test specimen study with more observers and a larger number of test specimens would be recommended in order to validate the results. Further test specimens could also be used to optimise the manufacturing process and would provide more images and thus more valid data for each X-ray setting being tested. Due to the larger data volume, it would also be possible to determine even better which test structures indicate changes in the X-ray system. A problem is going to be the increased workload during the evaluation process for the observer. Obviously, more X-ray images means a greater workload for the observers. This could lead to biases due to a lack of concentration during the scoring. This increased workload should be counteracted by prescribed short breaks for the observers while evaluating the X-ray images.

## Conclusions

A zoomorphic test specimen can be used for constancy testing of digital X-ray systems in veterinary medicine. Especially a lower dose can be recognised due to a deviation in the image quality on X-ray images of the test specimen when compared to the reference image. However, it is not possible to identify an overexposure with this method as the image quality (the noise impression in particular) was not scored better than in the reference image. The X-ray image of the test specimen shows good agreement with a latero-lateral thoracic image of a beagle. The test specimen manufactured using a 3D-printing method is relatively inexpensive compared to the test equipment used in human medicine.

## Data Availability

The datasets used and/or analysed during the current study are available from the corresponding author on reasonable request.
